# Editorial: The role of probiotics, postbiotics, and microbial metabolites in preventing and treating chronic diseases

**DOI:** 10.3389/fcimb.2023.1246937

**Published:** 2023-07-05

**Authors:** Tangchang Xu, Xia He, Tingtao Chen

**Affiliations:** ^1^ Department of Obstetrics and Gynecology, The Ninth Hospital in Nanchang and The Ninth Affiliated Hospital of Nanchang University, Nanchang, China; ^2^ School of Life Sciences, Nanchang University, Nanchang, China; ^3^ The Institute of Translational Medicine, Nanchang University, Nanchang, China

**Keywords:** probiotics, postbiotics, microbial metabolites, microbiome, multi-omics, chronic disease

Chronic diseases are defined as non-communicable diseases that are difficult to cure and cause host damage due to long-term accumulation, which mainly include cardiovascular and cerebrovascular diseases (hypertension, coronary heart disease, stroke, etc.), cancer, diabetes, and chronic obstructive pulmonary disease (chronic bronchitis, emphysema, etc.). Currently, there is no effective treatment methods for chronic diseases, and probiotics, postbiotics and microbial metabolites have become promising approaches for the improvement of chronic diseases.

In this Research Topic, Lv et al. conducted a clinical intervention experiment to investigate the therapeutic effects of precision probiotic strains transplantation capsules, fecal microbiota transplantation capsules and live combined *Bacillus subtilis* and *Enterococcus faecium* capsules on patients with severe diarrhea irritable bowel syndrome (IBS). Their results showed that precision probiotic strains transplantation capsules exhibited a more pronounced impact on IBS QoL, stool frequency, stool character, degree of abdominal pain, GAD-7 score and gut microbiota compared with other viable agents. Wu et al. explored the effect of dietary fiber on colitis using a mice model of antibiotic-induced *Clostridioides difficile* infection, their findings showed that different diets exerted varying effects on intestinal epithelial permeability, pectin consistently increased the diversity of the microbiome, and reduced the level of inflammation in serum and colonic tissue. Furthermore, the activator FICZ and the inhibitor CH2223191 of the aromatic hydrocarbon receptor (AhR) were used to confirm that pectin worked through the AhR signaling pathway. Similarly, Dong et al. discovered that sodium butyrate (NaB) and fecal microbiota transplantation (FMT) reversed ulcerative colitis to induce prostate enlargement, and up-regulated the expression of GPER and butyric acid in the prostate by a mouse model of ulcerative colitis (UC), suggesting that butyric acid and FMT may be potential treatments for UC-induced prostate enlargement. In addition, Hu et al. and Guo et al. made a comprehensive overview of the relationship between *Helicobacter pylori* and the development of gastric cancer (GC). In summary, *H. pylori* plays different roles in different stages of GC, and eradicating *H. pylori* and improving the diagnosis rate of early GC are effective ways to reduce the incidence of GC and improve the survival rate of GC.

For the brain diseases, Chen et al. predicted the poor outcome of acute ischemic stroke (AIS) with hyperlipidemia (POAH) patients and good outcome of AIS with hyperlipidemia (GOAH) patients by analyzing gut microbial characteristics. Their results indicated that there was a difference in the characteristics of the gut microbiota in POAH and GOAH, and that the relative abundance of *Enterococcaceae* and *Enterococcus* were enhanced, and *Lachnospiraceae*, *Faecalibacterium*, *Rothia* and *Butyricicoccus* were reduced. Besides, the receiver operating characteristic (ROC) model constructed based on gut microbiota can distinguish between POAH and GOAH, revealing that the microbial composition of POAH have a close correlation to clinical parameters, and the characteristics of gut microbiota can be used as important indicators for the diagnosis of POAH. Pan et al. studied the therapeutic effect and mechanism of probiotic *Pediococcus pentosaceus* on the mouse model of 1-methyl-4-phenyl-1,2,3,6-tetrahydropyridine (MPTP)-induced Parkinson’s disease (PD) by regulating the gut-brain axis. The results showed that *P. pentosaceus* could improve the movement disorder, dopaminergic neuronal degeneration, α-synuclein accumulation, significantly increase the levels of SOD1, Gpx1 and Nrf2 in the brain, reduce the level of Keap1, improve gut microbial dysbiosis, and increase GABA levels. These findings suggest that *P. pentosaceus* may be a promising candidate in the treatment of PD.

For other diseases, Qiao et al. explored the role of gut microbiota and fecal metabolites in the pathogenesis of disuse-induced osteoporosis (DIO), disclosing that gut microbiota and fecal metabolites may be potential factors leading to bone loss. Zhu et al. investigated the improvement of the extracts of the male zooid of *Antheraea pernyi* (EMZAP) on non-alcoholic fatty liver disease (NAFLD) induced by high-fat diet (HFD), indicating that EMZAP as a dietary supplement and functional food may improve HFD-induced NAFLD in the future. Tong et al. made a comprehensive overview of the effects of gut microbiota on the gout physiology and the effects of gout on gut microbiota, illustrating that prebiotics, probiotics, traditional Chinese medicine, and fecal transplantation may treat gout by changing the composition of the gut microbiota. Han et al. provided an overview of bacterial membrane vesicles in the pathophysiology of pneumonia and its complications. In general, targeting the key components of outer membrane vesicles that interact with human lung cells or macrophages may become a new strategy for the treatment of pneumonia in the future. Xie et al. comprehensively reviewed the role of *Lactobacillus* as a new therapeutic agent of atopic dermatitis (AD), suggesting that *Lactobacillus* may become a food supplement to improve AD. Furthermore, Pan et al. explored the regulation of tryptophan metabolism in lactic acid bacteria (LAB) by multi-omics methods, demonstrating that multi-omics technology will help to explore more LAB with tryptophan metabolic potential.

In conclusion, there are 13 papers in this Research Topic that provide a detailed overview of the potential roles of probiotics, postbiotics and microbial metabolites in the occurrence and development of various chronic diseases, which promote our understanding of the improvement of chronic diseases. In addition, we also hope that this Research Topic can help researchers put forward new strategies in the improvement of chronic diseases.

## Author contributions

All authors made a substantial, direct and intellectual contribution to this work, and approved it for publication.

